# Chronic high-fat feeding impairs adaptive induction of mitochondrial fatty acid combustion-associated proteins in brown adipose tissue of mice

**DOI:** 10.1016/j.bbrep.2017.02.002

**Published:** 2017-02-20

**Authors:** Takayuki Ohtomo, Kanako Ino, Ryota Miyashita, Maya Chigira, Masahiko Nakamura, Koji Someya, Niro Inaba, Mariko Fujita, Mitsuhiro Takagi, Junji Yamada

**Affiliations:** a Department of Pharmacotherapeutics, Tokyo University of Pharmacy and Life Sciences, 1432-1 Horinouchi, Hachioji, Tokyo 192-0392, Japan; b Center for the Advancement of Pharmaceutical Education, Tokyo University of Pharmacy and Life Sciences, 1432-1 Horinouchi, Hachioji, Tokyo 192-0392, Japan

**Keywords:** Acot, acyl-CoA thioesterase, Acox, acyl-CoA oxidase, BAT, brown adipose tissue, Cpt, carnitine palmitoyltransferase, ETC, electron transport chain, HFD and LFD, high- and low-fat diet, Ppar, peroxisome proliferator-activated receptor, SNS, sympathetic nervous system, TCA, tricarboxylic acid, TG, triglyceride, Ucp, uncoupling protein, WAT, white adipose tissue, Acyl-CoA thioesterase, Brown adipose tissue, Fatty acid oxidation, Obesity, Uncoupling protein

## Abstract

Since brown adipose tissue (BAT) is involved in thermogenesis using fatty acids as a fuel, BAT activation is a potential strategy for treating obesity and diabetes. However, whether BAT fatty acid combusting capacity is preserved in these conditions has remained unclear. We therefore evaluated expression levels of fatty acid oxidation-associated enzymes and uncoupling protein 1 (Ucp1) in BAT by western blot using a diet-induced obesity C57BL/6J mouse model. In C57BL/6J mice fed a high-fat diet (HFD) over 2–4 weeks, carnitine palmitoyltransferase 2 (Cpt2), acyl-CoA thioesterase (Acot) 2, Acot11 and Ucp1 levels were significantly increased compared with baseline and control low-fat diet (LFD)-fed mice. Similar results were obtained in other mouse strains, including ddY, ICR and KK-Ay, but the magnitudes of the increase in Ucp1 level were much smaller than in C57BL/6J mice, with decreased Acot11 levels after HFD-feeding. In C57BL/6J mice, increased levels of these mitochondrial proteins declined to near baseline levels after a longer-term HFD-feeding (20 weeks), concurrent with the accumulation of unilocular, large lipid droplets in brown adipocytes. Extramitochondrial Acot11 and acyl-CoA oxidase remained elevated. Treatment of mice with Wy-14,643 also increased these proteins, but was less effective than 4 week-HFD, suggesting that mechanisms other than peroxisome proliferator-activated receptor α were also involved in the upregulation. These results suggest that BAT enhances its fatty acid combusting capacity in response to fat overload, however profound obesity deprives BAT of the responsiveness to fat, possibly via mitochondrial alteration.

## Highlights

• BAT activation is a potential strategy for treating obesity and diabetes.
• BAT enhances its fatty acid combusting capacity in response to high-fat feeding.
• Profound obesity deprives BAT of the responsiveness to fat overload.
• Susceptibility to BAT activation could depend on the degree of obesity.

## 1 Introduction

Adult mammals have at least two types of adipocytes in the body: white adipocytes that accumulate fat as a triglyceride (TG) in cells in preparation for starvation, and brown adipocytes that combust fat to maintain body temperature. Brown adipocytes possess a high density of mitochondria in which energy derived from fatty acid degradation is dissipated as heat by the action of uncoupling protein 1 (Ucp1), which is exclusively expressed in this cell type [1]. For this reason, activation of brown adipocytes leads to an increase in calorie consumption and is expected to improve overweight conditions, providing a potential strategy for treating obesity and its related metabolic disorders [1], [2], [3], [4], [5]. In practice, metabolic activation of brown adipose tissue (BAT) has been shown in human subjects exposed to cold temperature by means of positron emission tomography with ^18^F-fluorodeoxyglucose (^18^F-FDG PET), and the ^18^F-FDG uptake is inversely correlated with body mass index and body fat percentage [5], [6]. Therefore, agents that could effectively stimulate BAT metabolic activity would be useful. However, whether BAT fatty acid combusting capacity is preserved in obese and diabetic conditions remains unclear.

To estimate BAT metabolic activity, non-invasive ^18^F-FDG PET is often used in humans, but ^18^F-FDG uptake is not an indicator specialized in fatty acid combustion. In rodent experiments, Ucp1 expression level has primarily been measured, because Ucp1 is central in the mechanism for energy expenditure in brown adipocytes. However, Ucp1 level does not exactly reflect fatty acid combusting capacity, and results reported in previous studies have not been consistent. For example, while BAT Ucp1 expression level was lower in obese ob/ob mice compared with normal mice [7], it was higher in obese mice fed a high-fat diet (HFD) than in control low-fat diet (LFD)-fed mice in most cases, with a huge variation in magnitude from negligible to remarkable levels [2], [8], [9]. In some cases, HFD had no effect or even lowered Ucp1 expression [8]. These differences are possibly due to differences in duration of HFD-feeding period and/or Ucp1 estimation based on protein or mRNA levels, as well as variations in genetic- or diet-induced obesity. Moreover, few studies have evaluated fatty acid oxidation along with Ucp1 in BAT of obese or diabetic animals [9].

In this study, we examined the changes in fatty acid combusting capacity by measuring protein expression levels of fatty acid oxidation-associated enzymes, including carnitine palmitoyltransferase 2 (Cpt2), acyl-CoA thioesterase 2 (Acot2), Acot11 and acyl-CoA oxidase 1 (Acox1), as well as Ucp1 and peroxisome proliferator-activated receptor (Ppar) γ in BAT, using a diet-induced obesity mouse model established by HFD-feeding. Our results suggest that profound obesity induced by chronic high-fat feeding could impair the adaptive induction of fatty acid combustion in BAT mitochondria.

## 2 Materials and methods

This study included adult male C57BL/6J mice (the strain which is most widely used in obesity research), ddY mice (as a model of postprandial hypertriglyceridemia) [10], KK-*A*^*y*^ (a model of type 2 diabetes) [11] and ICR (control) mice. All animals were conditioned to an environment at 23±1 °C with constant humidity of 55±5% and a 12 h light/12 h dark cycle and given free access to food and tap water according to the Guide for the Care and Use of Laboratory Animals published by the US National Institute of Health (NIH Publication No. 85-23, revised 1996). The protocol of this study was approved by the Committee of Animal Use and Welfare of Tokyo University of Pharmacy and Life Sciences.

### 2.1 Animals and treatment

Male C57BL/6J, ddY and ICR mice (Tokyo Laboratory Animals Science, Tokyo, Japan) and KK-A^y^ mice (CLEA Japan, Tokyo, Japan) were acclimatized at the age of 9 weeks for 1 week on a LFD (D12450B; Research Diets, New Brunswick, NJ, U.S.A) and subsequently fed a LFD or HFD (D12492; Research Diets) for 2, 4 or 20 weeks ad libitum. The LFD contained 3.85 kcal/g, with percentages of carbohydrate, fat and protein of 70%, 10% and 20%, respectively. The HFD contained 5.24 kcal/g with percentages of 20%, 60% and 20%, respectively. The carbohydrate was a combination of cornstarch, maltodextrin and sucrose, while the fat was soybean oil and lard. A group of C57BL/6J mice were orally administered Wy-14,643 (Tokyo Chemical Industry, Tokyo, Japan) at 50 mg/kg, once a day for 2 weeks after acclimatization, and were maintained on a LFD. After an overnight fast, the mice were anesthetized by intraperitoneal injection of a mixture of medetomidine (0.75 mg/kg), midazolam (4 mg/kg) and butorphanol (5 mg/kg), and sacrificed by decapitation. The serum was collected, and interscapular BAT and other tissues were excised, snap-frozen and stored at −80 °C until analysis.

### 2.2 Preparation of BAT homogenates

BAT homogenates were prepared in 250 mM sucrose containing 1 mM EDTA, 10 mM Tris-HCl (pH 7.5) and Complete protease inhibitor cocktail (Sigma-Aldrich, St. Louis, MO, USA) using a Potter-Elvehjem glass homogenizer with a glass pestle. After the homogenates were centrifuged at 25,000*g* for 10 min at 4 °C, the semisolid fat cake floating on the surface was removed on ice and the remaining portion was rehomogenized for use. The protein concentrations were determined using a DC protein assay kit (Bio-Rad, Hercules, CA, USA) with bovine serum albumin as the standard.

### 2.3 Western blotting

Proteins were resolved in 10% SDS-polyacrylamide gels and immunoreactivity was detected by ImmunoStar LD reagent (Wako Pure Chemical Industries, Osaka, Japan) using a luminoimaging analyzer, as described previously [12]. For quantitative analyses, the signal intensities of the bands detected on the membranes were measured and transformed into relative values using a calibration curve generated with known amounts of protein. To detect Acot11, a rabbit polyclonal antibody against a polypeptide corresponding to mouse Acot11 amino acids 601–614 (H_2_N-CLDNRNDLAPSLQTL-CONH_2_) was raised and affinity-purified as described previously [12]. Rabbit polyclonal antibodies against Cpt2, Acot1 (which cross-reacts with Acot2) and Acox1 were described in our previous studies [13], and those against Ucp1 (AnaSpec, Fremont, CA, USA) and Pparγ (Santa Cruz Biotechnology, Dallas, TX, USA) were obtained from commercial sources.

### 2.4 Oil Red O staining

BAT specimens were fixed with 4% paraformaldehyde, rinsed with water, transferred into 30% sucrose in PBS at 4 °C until specimens sunk, and embedded in Optimal Cutting Temperature (OCT) matrix compound. OTC-embedded specimens were cut into 4 µm-thick sections and stained for 60 min at 37 °C in Oil red O solution followed by counterstaining with hematoxylin and eosin. Oil red O staining was carried out by Biopathology Institute Co., Ltd. (Oita, Japan). Microscopic examinations were performed using a BZ-8100 microscope (Keyence, Osaka, Japan).

### 2.5 Statistical analysis

The statistical significance of differences among values was examined by two-way factorial analysis of variance followed by Bonferroni's multiple comparison tests. Values of *p*<0.05 were considered to indicate statistical significance. Statistical analyses were performed with Prism 7.0 for Mac (GraphPad, La Jolla, CA, USA).

## 3 Results

**Fig. 1:**
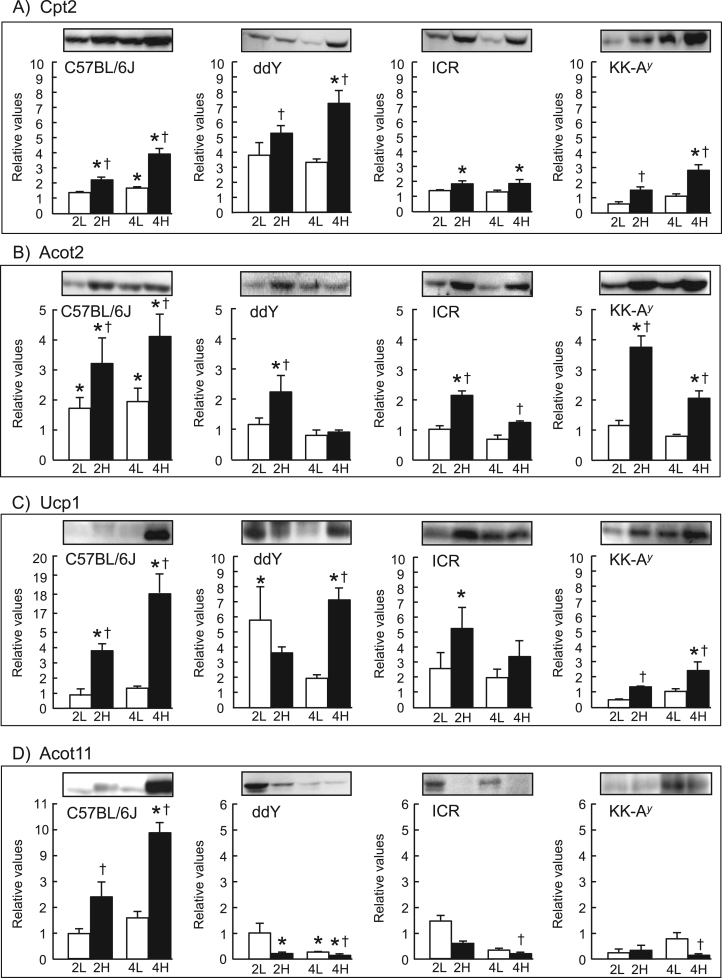
Changes in expression levels of Cpt2 (A), Acot2 (B), Ucp1 (C) and Acot11 (D) in BAT of mice after short-term HFD-feeding. After 1 week of acclimatization (baseline), different strains of mice (C57BL/6J, ddY, ICR and KK-A^y^) were fed a LFD for 2 (2 L) or 4 weeks (4 L), or a HFD for 2 (2 H) or 4 weeks (4 H), and the expression levels of the proteins (A–D) were analyzed by western blotting with BAT homogenates. Signal intensities of the protein bands (per mg protein) were measured and are expressed relative to the baseline values, which were set as 1 (mean±SEM of 3–7 mice). *p<0.05 *vs.* baseline; †*p*<0.05 *vs.* the LFD group at the same feeding time period. Representative western blots (20 µg protein/lane) for Cpt2 (65 kDa), Acot2 (45 kDa), Ucp1 (30 kDa) and Acot11 (65 kDa) are shown.

We first examined the effect of short-term HFD-feeding (for up to 4 weeks) on fatty acid combustion-associated proteins in BAT of C57BL/6J mice (Fig. 1). These protein levels were already elevated after 2 weeks, and significantly higher (*p*<0.05) than the respective baseline and control levels seen in BAT of LFD-fed mice after 4 weeks. Body weight gain and white adipose tissue (WAT) weights, BAT weight, and serum levels of alanine aminotransferase (ALT) and glucose of HFD-fed mice were higher than those of LFD-fed mice, confirming establishment of the HFD-induced obesity model (Table S1).

Similar results were obtained for Cpt2, Acot2 and Ucp1 in BAT of ddY, ICR and genetically diabetic KK-A^y^ mice, although the extent of increase in the protein levels varied among mouse strains, with the most consistent induction observed in C57BL/6J mice (Fig. 1). Greater body weight gains in HFD-fed ddY, ICR and KK-A^y^ mice and higher serum glucose level in HFD-fed KK-A^y^ mice than in the respective LFD-fed control mice were also confirmed (Tables S2–4). However, Acot11 was increased in C57BL/6J mice, but decreased in the other three mouse strains. Moreover, basal levels of Ucp1 and Acot11 were 2–4 times higher in BAT of KK-A^y^ mice compared with the other strains (Fig. S1).

**Fig. 2:**
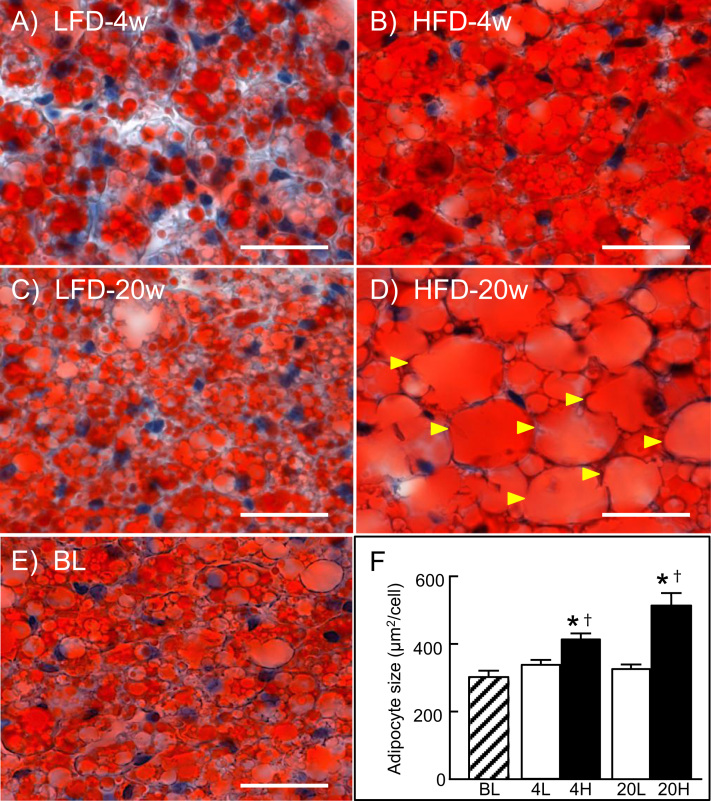
Oil Red O staining of BAT sections. Sections were prepared from C57BL/6J mice fed a LFD for 4 (A) or 20 weeks (C), or a HFD for 4 (B) or 20 weeks (D), or baseline (E). In D, arrowheads indicate cells containing unilocular large lipid droplet. Scale bars, 20 µm. (F) Quantitative analysis of adipocyte size, expressed in µm^2^. Areas were calculated from 50 adipocytes of 5–8 mice in each group. Result are mean±SEM. *p<0.05 vs. baseline; †*p*<0.05 vs. the LFD group at the same feeding time period.

**Fig. 3:**
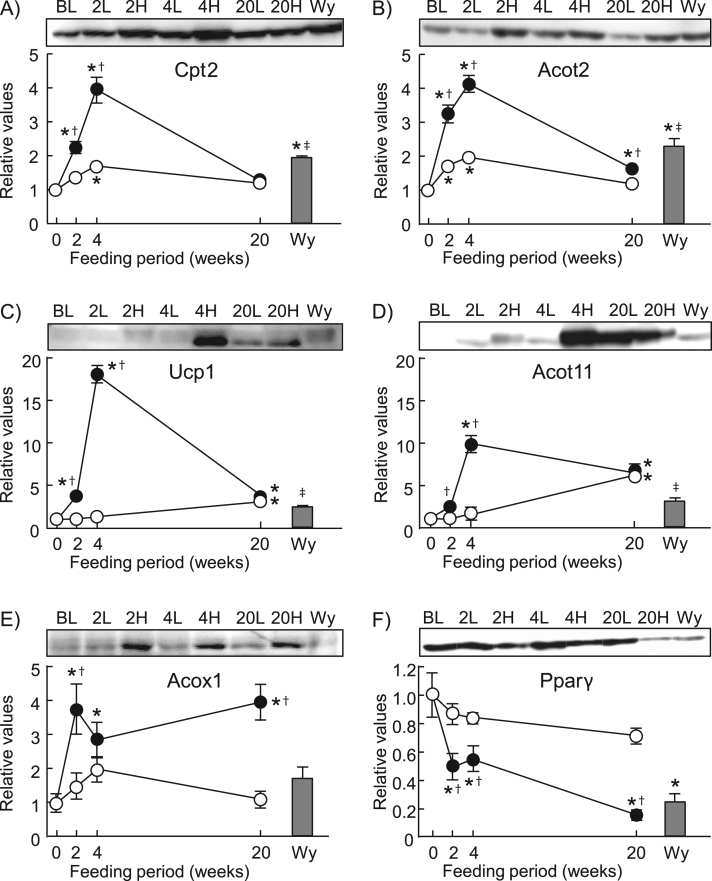
Changes in expression levels of Cpt2 (A), Acot2 (B), Ucp1 (C), Acot11 (D), Acox1 (E) and Pparγ (F) in BAT of mice after long-term HFD-feeding. After 1 week of acclimatization [baseline (BL)], C57BL/6J mice were fed a LFD (open circles) or HFD (closed circles) for up to 20 weeks. A group of mice were treated with Wy-14,643 for 2 weeks (Wy) (shaded columns), being maintained on the LFD. The expression levels of the proteins (A–F) were analyzed by western blotting with BAT homogenates. Signal intensities of the protein bands (per mg protein) were measured and are expressed relative to the baseline values, which were set as 1 (mean±SEM of 5–8 mice). *p<0.05 *vs.* BL; †*p*<0.05 *vs.* the LFD group at the same feeding time period; ‡*p*<0.05 between Wy group and HFD group at 4 week-feeding. Representative western blots (20 µg protein/lane) are also shown for Cpt2 (65 kDa), Acot2 (45 kDa), Ucp1 (30 kDa), Acot11 (65 kDa), Acox1 (52 kDa subunit) and Pparγ (52 kDa) in BAT of mice fed LFD for 2, 4 or 20 weeks (2 L, 4 L and 20 L, respectively), or HFD for 2, 4 or 20 weeks (2 H, 4 H and 20 H, respectively). The data shown in Fig. 1 for Cpt2, Acot2, Ucp1 and Acot11 of C57BL/6J mice are plotted as the corresponding values in A–D, respectively.

We next examined the effect of long-term HFD-feeding in C57BL/6 J mice. In mice fed HFD for 20 weeks, body weight gain, WAT weights, BAT weight, liver weight, and serum levels of ALT and glucose were significantly higher (*p*<0.05) than LFD-fed control mice and baseline levels (Table S5). In BAT examined by microscopy after Oil Red O staining, unilocular large lipid droplets accumulated within brown adipocytes of these mice (Fig. 2D), whereas multimolecular large lipid droplets (Fig. 2B) and small lipid droplets (Fig. 2A and C) were observed in mice fed HFD for 4 weeks and mice fed LFD, respectively. In BAT of the mice fed HFD for 20 weeks, Cpt2, Acot2 and Ucp1 levels were the same or nearly the same as those seen in the control LFD-fed mice and baseline levels, respectively (Fig. 3). In contrast, Acot11 level was 7 times higher than the baseline, although it was similar in HFD- and LFD-fed mice. Moreover, Acox1 increased on a short-term HFD and remained elevated after long-term HFD feeding at a level about 4 times higher than the control and baseline levels.

To examine a possible involvement of Pparα, a nuclear receptor regulating expression of fatty acid oxidation-associated enzymes [14], [15], C57BL/6J mice fed on LFD were treated with Wy-14,643, a synthetic ligand specific to Pparα, for 2 weeks (Fig. 3). In these mice, Cpt2 and Acot2 expressions were markedly upregulated in the liver, confirming the effectiveness of this agent to induce these enzymes (Fig. S3). However, it was much less effective in upregulating expression of Cpt2, Acot2, Ucp1, Acot11 and Acox1 in BAT compared with levels in 4 week-HFD mice. Pparγ level in BAT declined by about 50% over 2–4 weeks and by more than 80% from the baseline after 20 weeks of HFD-feeding, whereas it was almost sustained on LFD (Fig. 3F). Correlation coefficient analysis among the 6 proteins examined in BAT of HFD-fed C57BL/6J mice (data shown in Fig. 3) indicated that the expressions of mitochondria-localized proteins Cpt2, Acot2 and Ucp1 were significantly intercorrelated (*p*<0.001) (Fig. S2). Cpt2 and Acot2 were also correlated with Pparγ. Acot11 and Acox1 were not correlated with any other proteins (*p*>0.05).

## 4 Discussion

**Fig. 4:**
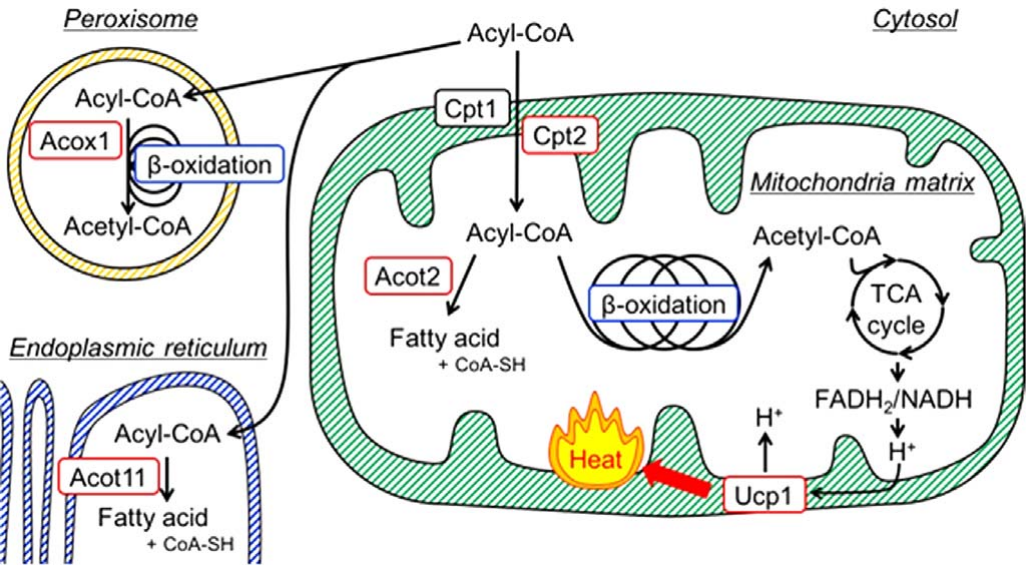
In brown adipocytes, acyl-CoA is transported via Cpt (Cpt1 and Cpt2) to the mitochondria matrix. Acot2 is induced along with β-oxidation enzymes and hydrolyzed some of acyl-CoA to generate fatty acid and CoA-SH. Thus, upregulation of Acot2 serves to maintain an adequate rate of β-oxidation by modulating the substrate supply and retaining the level of coenzymes used in the TCA cycle and β-oxidation itself. Ucp1 releases the energy extracted from fatty acids via β-oxidation followed by TCA cycle as heat (i.e. fatty acid combustion) by uncoupling ETC from the oxidative phosphorylation. These proteins (Cpt2, Acot2 and Ucp1) contribute to efficient oxidation of fatty acids in mitochondria. Acox1 oxidizes fatty acid to promote β-oxidation in peroxisome. Acot11 hydrolyzes some acyl-CoA to fatty acid and CoA-SH in the endoplasmic reticulum. Acot, acyl-CoA thioesterase; Acox1, acyl-CoA oxidase; Cpt, carnitine palmitoyltransferase; ETC, electron transport chain; TCA, tricarboxylic acid; Ucp, uncoupling protein.

Our model for the roles of Cpt2, Acot2, Acot11, Acox1 and Ucp1 in BAT is shown in Fig. 4. We demonstrated that Cpt2, Acot2 and Ucp1 protein levels significantly increased in BAT of mice on a short-term HFD-feeding, but the increased proteins levels were not sustained and returned to basal or nearly basal levels after prolonged HFD-feeding. These results suggested that in response to fat overload, fatty acid oxidation was upregulated along with energy-dissipating Ucp1 activity in mitochondria of brown adipocytes. However, this adaptive induction of fatty acid combusting capacity of BAT was impaired in profound obese and diabetic conditions.

### 4.1 Short-term HFD-feeding

In this study, we used Cpt2 and Acot2 as markers for mitochondrial fatty acid oxidation. Cpt2 is involved in uptake of long-chain acyl-CoAs into the mitochondrial matrix, in which Acot2 modulates supply of acyl-CoA substrates to β-oxidation system by hydrolyzing long-chain acyl-CoA thioesters to free fatty acids and CoA-SH. Although the role of Acot2 remains to be fully established, its close relationship with fatty acid oxidation has been repeatedly described in rodent experiments, because Acot2 is expressed in highly oxidative tissues such as cardiac and skeletal muscles and is upregulated by fasting, diabetic conditions and synthetic Pparα ligands in the liver and kidney [16], [17], [18], [19]. Significant levels of Acot2 have also been demonstrated in BAT [20]. In our previous study, Acot2 and Cpt2 were co-induced during maturation of cultured rat brown adipocytes after differentiation of preadipocytes, and then upregulated by feeding cells on palmitate [21]. These findings provide an idea that Acot2 may constrain the β-oxidation rate by limiting substrate supply and maintain the level of coenzymes used in the tricarboxylic acid (TCA) cycle and β-oxidation to support effective fatty acid oxidation, especially when acyl-CoA is overloaded into the mitochondrial matrix where enhanced β-oxidation cannot be accompanied by appropriate upregulation of the TCA cycle or electron transport chain (ETC) activity [16], [21]. Consistent with this idea, a recent study using a model of adenoviral Acot2 overexpression in mouse liver also indicated that Acot2 can facilitate mitochondrial fatty acid oxidation [22]. Therefore, it is reasonable that Acot2 was adaptively upregulated along with Cpt2 in BAT against fatty acids overloaded by HFD-feeding in the short term, as was the case in cardiac and skeletal muscles where fatty acids should be preferentially used as fuel [13], [18], [19].

While energy derived from fatty acid oxidation undergoes a chemical conversion to ATP, which is largely consumed by contractile activity in muscles, it can be released as heat via its uncoupling from oxidative phosphorylation in brown adipocytes, leading to non-shivering thermogenesis [1]. However, thermogenesis in BAT is a physiological function to maintain body temperature in the face of a cold environment, but not to dissipate excess energy from dietary fat intake so as to maintain optimal body weight, and therefore diet-induced (metaboloregulatory) thermogenesis by BAT is considered unlikely [23]. Nevertheless, our results demonstrated that Ucp1 level significantly increased in concert with upregulation of Cpt2 and Acot2 in BAT of mice after short-term HFD-feeding. In case of cold-induced (thermoregulatory) thermogenesis, chronic adrenergic stimulation by the activated sympathetic nervous system (SNS) promotes total capacity of BAT, a process referred to as “recruitment” [1], [2], [3]. It is unclear whether the HFD-feeding for 2–4 weeks led to increased SNS activity, but HFD, albeit for a few days, does not raise SNS activity in BAT of mice, unlike in muscle [24].

Although we did not examine thermogenesis, the mitochondrial fatty acid combusting capacity in BAT was suggested to be upregulated. This was basically the case with different mouse strains, even with diabetic KK-A^y^ mice. To get a clue about the underlying mechanisms for the upregulation, we tried Wy-14,643 as a synthetic ligand specific to Pparα, but it was much less effective. In our previous study using cultured rat brown adipocytes, Wy-14,643 also had no significant effect on Cpt2 and Acot2 expression levels [21]. This finding indicates that the low levels of effectiveness of Wy-14,643 observed in this study was due to the intrinsic characteristics of brown adipocytes, and not from the drug delivery to BAT in mice. This suggests that mechanisms other than Pparα were also involved in the HFD-induced upregulation of Cpt2, Acot2 and Ucp1 in mouse BAT.

### 4.2 Mouse strain-associated differences

Acot family members exhibit various cellular and subcellular localizations, with overlaps among the isoforms [16], [17]. Acot11 is almost exclusively expressed in BAT and largely concentrated in the endoplasmic reticulum [25], [26], [27], [28]. Acot11 was recently reported to limit the oxidation of lipid droplet-derived fatty acids, possibly by regulating the availability of substrates to be used for β-oxidation and uncoupling [26], [27], [28]. In this study we revealed significant upregulation of Acot11 after 4-week HFD-feeding in C57BL/6J mice. However, it was downregulated in ddY, ICR and KK-A^y^ mice under the same conditions. Moreover, the basal level of Acot11 in BAT of KK-A^y^ mice, together with Ucp1, was several times higher than those of the other mice. At present, the reasons for these differences are unclear, but it would be of interest to analyze the genetic backgrounds implicated in these mouse strain-associated differences in Acot11 expression to gain an insight into the basis of BAT activity variation in human individuals.

### 4.3 Long-term HFD-feeding

The reason why the HFD-induced upregulation of Cpt2, Acot2 and Ucp1 was impaired by long-term HFD-feeding may be explainable in terms of “BAT whitening”. BAT whitening is associated with an accumulation of enlarged lipid droplets and mitochondrial dysfunction and loss in brown adipocytes under overnutrition, giving the tissue a WAT-like phenotype [29]. In the mice fed HFD for 20 weeks, their obesity and diabetic conditions became exacerbated as seen by elevated values of body, WAT and liver weights, serum glucose and ALT, and large unilocular lipid droplets characteristic to white adipocytes that accumulated in the brown adipocytes, concurrent with increased BAT weight. These results suggested that the BAT whitened to acquire a WAT-like phenotype, including reduced activity of fatty acid oxidation. In practice, the increased expression of mitochondria-localized proteins, Cpt2, Acot2 and Ucp1, declined in an intercorrelated manner. The level of Pparγ, a nuclear receptor that plays a role in promoting Ucp1 expression and mitochondriogenesis in brown adipocytes [30], [31], also decreased in a similar manner, whereas extramitochondrial Acot11 and Acox1, a peroxisomal β-oxidation-associated enzyme, did not.

However, why the increased mitochondrial proteins did not fall below the basal and LFD (control) levels after long-term HFD was less clear. In a previous study, Ucp1 mRNA level decreased to nearly half the LFD level in whitened BAT of C57BL/6 mice fed a HFD for 8 weeks [27]. It is unclear whether the adaptive induction elicited earlier still continued to antagonize the whitening during a period as long as 20 weeks. Another plausible hypothesis may be that despite the upregulation of Acot2, prolonged fatty acid overload could impair the balance between β-oxidation and TCA cycle/ETC activity, resulting in a deposit of incomplete fat catabolites along with diminished levels of TCA cycle intermediates [13], [16], [32], [33]. These stressful environments created within mitochondria would possibly damage its function via enhanced oxidative stress, for example. After fatty acids are taken up into brown adipocytes, a portion of them should be burned off (immediately or after storage as TG), and another portion should accumulate in lipid droplets under overnutrition. Thus, both the mitochondrial stress and whitening could synergistically impair the adaptive induction of fatty acid combusting capacity of BAT when fats are chronically overloaded.

### 4.4 Conclusion

In the BAT of mice, at least two states exist; one allows the fat combusting capacity to increase against HFD-feeding, and the other does not. These two states should represent distinct aspects that constitute a continuous sequence in responsiveness of brown adipocytes to fat overload, which becomes impaired as adiposity progresses. This concept should be taken into consideration when a diet-induced obesity mouse model is used in studies. If this is also the case with human obese subjects, much attention should also be given when evaluating the efficacy of agents that are expected to activate BAT and thereby consume taken-up dietary calories to improve overweight conditions. The varying states of brown adipocytes could create a considerable difference in susceptibility to these agents among individuals, depending on their degree of obesity.

## Funding

This research did not receive any specific grant from funding agencies in the public, commercial, or not-for-profit sectors.
