# Supplementary material for: Chronic high-fat feeding impairs adaptive induction of mitochondrial fatty acid combustion-associated proteins in brown adipose tissue of mice
Source: Biochem Biophys Rep. 2017 Feb 20;10:32–8. doi: 10.1016/j.bbrep.2017.02.002 (PMC5614659; doi:10.1016/j.bbrep.2017.02.002)
Supplement: Table S2 — Supplementary material [file mmc2.pptx]

## Slide 1
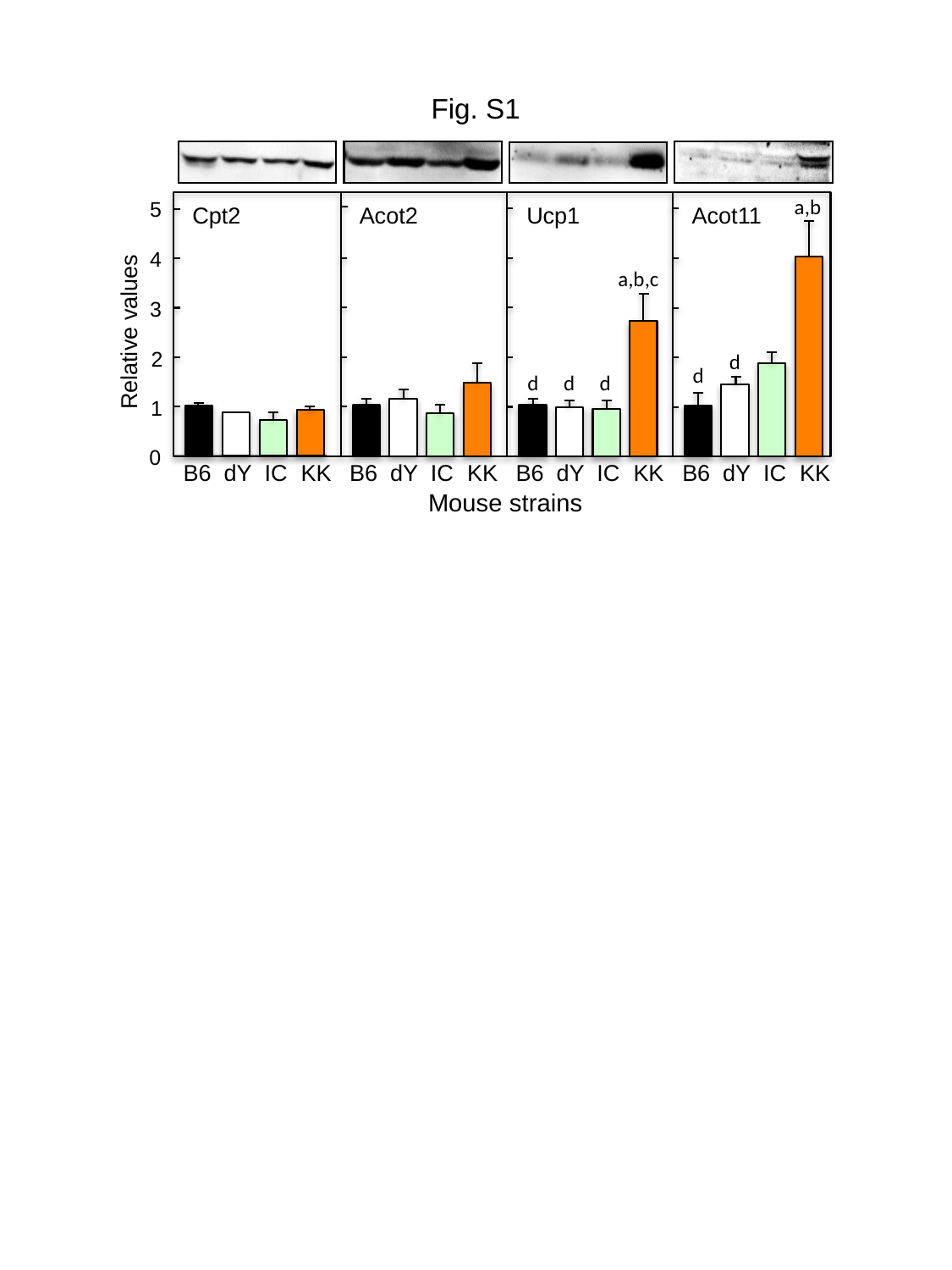

Fig. S1
a,b
5
4
3
2
1
0
Cpt2
Acot2
Ucp1
Acot11
a,b,c
Relative values
d
d
d
d
d
KK
IC
dY
B6
KK
IC
dY
B6
KK
IC
dY
B6
KK
IC
dY
B6
Mouse strains
