# Supplementary material for: Chronic high-fat feeding impairs adaptive induction of mitochondrial fatty acid combustion-associated proteins in brown adipose tissue of mice
Source: Biochem Biophys Rep. 2017 Feb 20;10:32–8. doi: 10.1016/j.bbrep.2017.02.002 (PMC5614659; doi:10.1016/j.bbrep.2017.02.002)
Supplement: Table S3 — Supplementary material [file mmc3.pptx]

## Slide 1
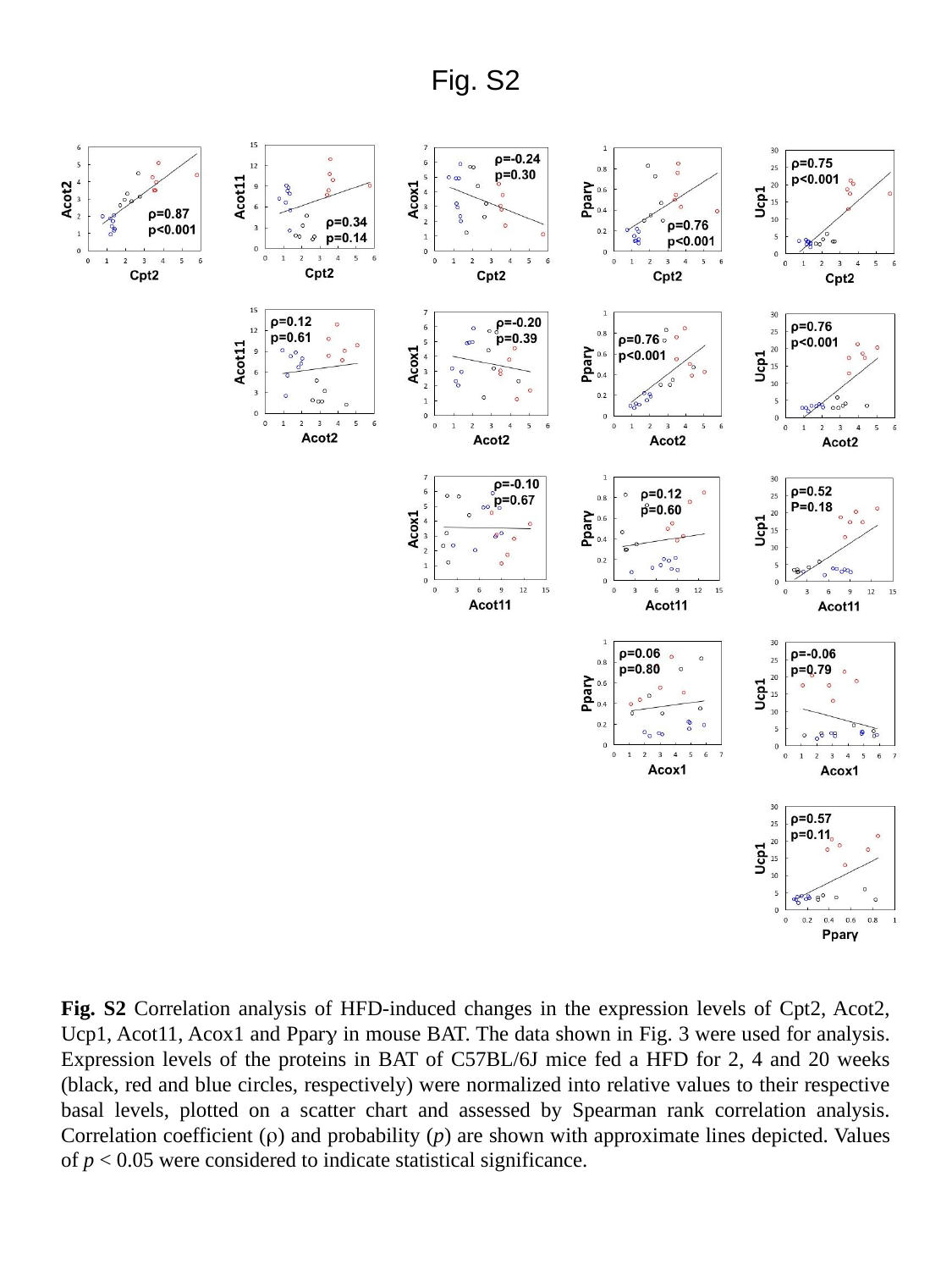

Fig. S2
Fig. S2 Correlation analysis of HFD-induced changes in the expression levels of Cpt2, Acot2, Ucp1, Acot11, Acox1 and Pparg in mouse BAT. The data shown in Fig. 3 were used for analysis. Expression levels of the proteins in BAT of C57BL/6J mice fed a HFD for 2, 4 and 20 weeks (black, red and blue circles, respectively) were normalized into relative values to their respective basal levels, plotted on a scatter chart and assessed by Spearman rank correlation analysis. Correlation coefficient (r) and probability (p) are shown with approximate lines depicted. Values of p < 0.05 were considered to indicate statistical significance.
