# Supplementary material for: Chronic high-fat feeding impairs adaptive induction of mitochondrial fatty acid combustion-associated proteins in brown adipose tissue of mice
Source: Biochem Biophys Rep. 2017 Feb 20;10:32–8. doi: 10.1016/j.bbrep.2017.02.002 (PMC5614659; doi:10.1016/j.bbrep.2017.02.002)
Supplement: Table S4 — Supplementary material [file mmc4.pptx]

## Slide 1
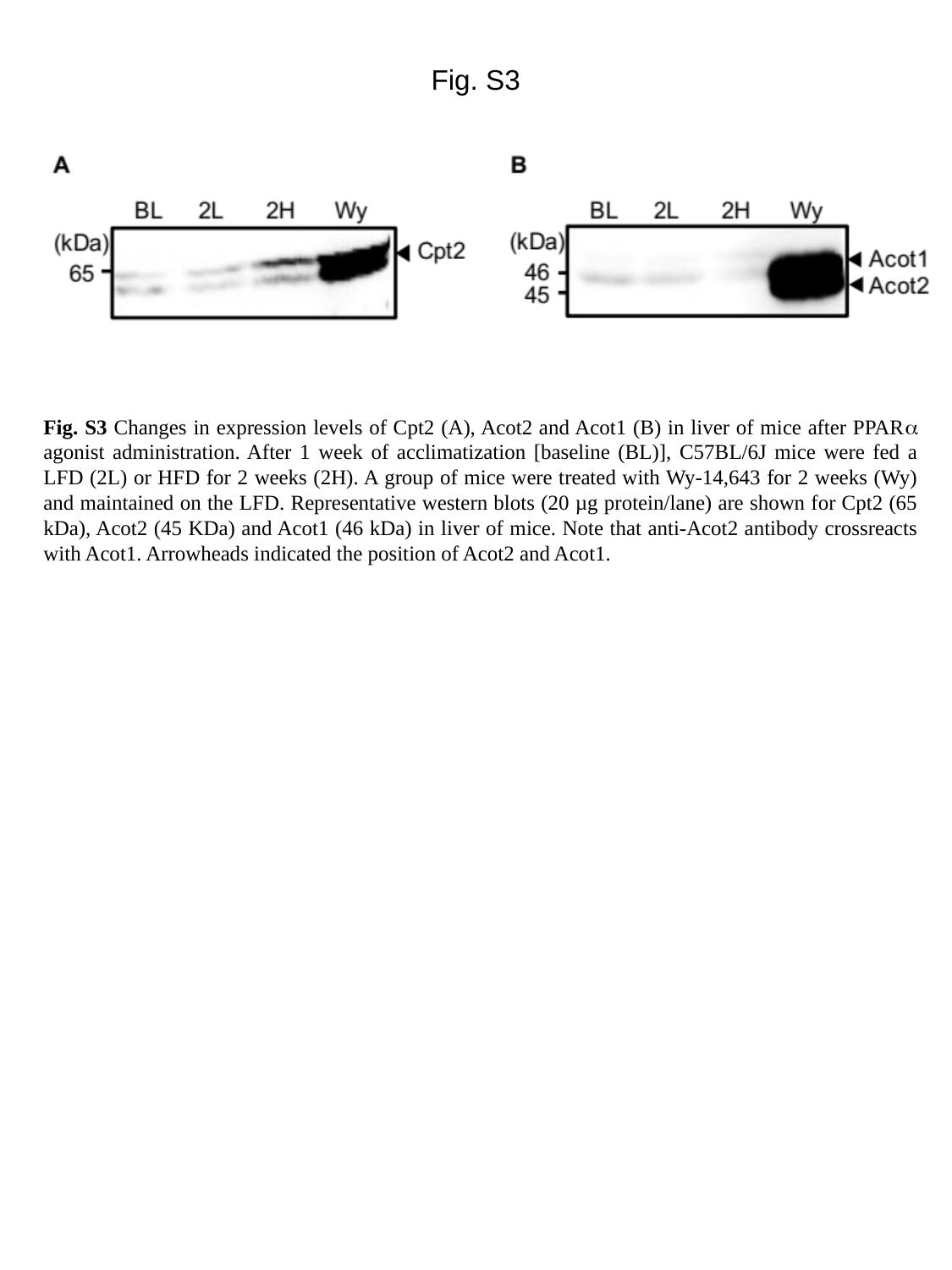

Fig. S3
Fig. S3 Changes in expression levels of Cpt2 (A), Acot2 and Acot1 (B) in liver of mice after PPARa agonist administration. After 1 week of acclimatization [baseline (BL)], C57BL/6J mice were fed a LFD (2L) or HFD for 2 weeks (2H). A group of mice were treated with Wy-14,643 for 2 weeks (Wy) and maintained on the LFD. Representative western blots (20 µg protein/lane) are shown for Cpt2 (65 kDa), Acot2 (45 KDa) and Acot1 (46 kDa) in liver of mice. Note that anti-Acot2 antibody crossreacts with Acot1. Arrowheads indicated the position of Acot2 and Acot1.
