# Supplementary material for: Chronic high-fat feeding impairs adaptive induction of mitochondrial fatty acid combustion-associated proteins in brown adipose tissue of mice
Source: Biochem Biophys Rep. 2017 Feb 20;10:32–8. doi: 10.1016/j.bbrep.2017.02.002 (PMC5614659; doi:10.1016/j.bbrep.2017.02.002)
Supplement: Table S5 — Supplementary material [file mmc5.pdf]

Table S1
Body weight gains, tissue weights, and serum nutritional and biochemical parameters of HFD-fed C57BL/6J mice.

C57BL/6J	Baseline	2 weeks		4 weeks	
		LFD	HFD	LFD	HFD
Body weight gain (g)	–	2.8±0.2	5.7±0.4†	1.9±0.4	8.9±0.8†
eWAT weight (g)	0.28±0.01	0.39±0.04	0.87±0.14*†	0.46±0.03	1.37±0.12*†
rWAT weight (g)	0.06±0.00	0.10±0.01	0.28±0.04*†	0.11±0.01	0.41±0.03*†
BAT weight (g)	0.07±0.01	0.06±0.06	0.06±0.00	0.06±0.00	0.07±0.00†
Liver weight (g)	0.90±0.01	0.93±0.04	1.00±0.04	0.91±0.02	1.00±0.04
Serum level of					
AST (IU/L)	36.2±3.9	45.6±10.8	59.1±12.9	44.3±9.2	50.6±8.5
ALT (IU/L)	12.7±3.6	8.9±1.4	10.0±1.3	6.0±0.8	11.6±1.7†
Glucose (mg/dL)	113.5±5.0	115.8±3.2	139.4±16.2	123.0±7.1	167.3±11.9*†
TG (mg/dL)	118.9±16.7	91.2±8.7	81.6±12.1	69.0±5.9*	77.0±7.0*
FFA (mEq/dL)	1.40±0.08	1.33±0.08	1.23±0.08	1.42±0.08	1.15±0.07†

Data represent means ± SEM (9–15 mice). *$p < 0.05$ *vs.* baseline; †$p < 0.05$ *vs.* the LFD group at the same feeding time period. eWAT, epididymal WAT; rWAT, retroperitoneal WAT; AST, aspartate transaminase; ALT, alanine transaminase; FFA, free fatty acid.

Table S2
Body weight gains, tissue weights, and serum nutritional and biochemical parameters of HFD-fed ddY mice.

ddY	Baseline	2 weeks		4 weeks	
		LFD	HFD	LFD	HFD
Body weight gain (g)	–	0.7±0.1	4.9±0.8†	2.4±0.1	10.2±0.7†
eWAT weight (g)	0.56±0.05	0.56±0.35	1.70±0.27*	1.01±0.33	2.43±0.45*†
rWAT weight (g)	0.21±0.04	0.15±0.10	0.57±0.10	0.28±0.08	0.73±0.14*†
BAT weight (g)	0.15±0.02	0.09±0.02	0.14±0.02	0.17±0.03	0.19±0.03
Liver weight (g)	1.38±0.05	1.39±0.06	1.49±0.07	1.34±0.12	1.49±0.07
Serum level of					
AST (IU/L)	45.1±5.9	46.6±5.1	40.1±5.9	49.0±24.2	29.3±2.2
ALT (IU/L)	9.9±0.3	12.3±3.5	10.8±0.9	7.2±1.9	10.0±0.6
Glucose (mg/dL)	105.3±13.6	142.0±17.8	112.3±16.5	130.7±1.5	172.5±24.4*
TG (mg/dL)	105.2±20.5	62.1±27.2	74.2±10.3	85.7±27.2	71.0±9.1
FFA (mEq/dL)	1.29±0.16	0.97±0.19	1.08±0.15	1.14±0.14	1.12±0.13

Data represent means ± SEM (3–4 mice). *$p < 0.05$ *vs.* baseline; †$p < 0.05$ *vs.* the LFD group at the same feeding time period. eWAT, epididymal WAT; rWAT, retroperitoneal WAT; AST, aspartate transaminase; ALT, alanine transaminase; FFA, free fatty acid.

Table S3
Body weight gains, tissue weights, and serum nutritional and biochemical parameters of HFD-fed ICR mice.

ICR	Baseline	2 weeks		4 weeks	
		LFD	HFD	LFD	HFD
Body weight gain (g)	–	3.0±0.3	3.4±0.7	3.6±0.4	6.1±1.0†
eWAT weight (g)	0.77±0.09	0.64±0.14	1.48±0.35	0.92±0.26	1.09±0.33*
rWAT weight (g)	0.24±0.04	0.18±0.04	0.51±0.10*†	0.25±0.07	0.41±0.07*†
BAT weight (g)	0.14±0.01	0.11±0.01	0.11±0.01	0.19±0.03*	0.11±0.02
Liver weight (g)	1.47±0.04	1.62±0.07	1.55±0.04	1.52±0.07	1.57±0.09
Serum level of					
AST (IU/L)	32.9±3.0	43.2±7.6	59.2±16.4	25.9±2.3	38.7±7.3
ALT (IU/L)	8.0±0.9	10.5±2.6	20.1±6.6	17.4±8.4	12.9±2.4
Glucose (mg/dL)	107.0±5.9	104.7±10.8	151.8±12.4	120.7±6.4	131.0±20.2
TG (mg/dL)	125.2±15.6	96.1±25.1	62.5±11.9*	77.5±15.2	57.4±10.1*
FFA (mEq/dL)	1.53±0.10	1.39±0.19	1.13±0.13*	1.12±0.08*	0.95±0.10*

Data represent means ± SEM (6–7 mice). *$p < 0.05$ *vs.* baseline; †$p < 0.05$ *vs.* the LFD group at the same feeding time period. eWAT, epididymal WAT; rWAT, retroperitoneal WAT; AST, aspartate transaminase; ALT, alanine transaminase; FFA, free fatty acid.

Table S4

Body weight gains, tissue weights, and serum nutritional and biochemical parameters of HFD-fed KK-A^y mice.

KK-A^y	Baseline	2 weeks		4 weeks	
		LFD	HFD	LFD	HFD
Body weight gain (g)	–	4.1±0.6	9.7±0.9†	7.9±0.7	12.2±1.5†
eWAT weight (g)	0.89±0.20	1.26±0.04	1.77±0.14*†	1.39±0.10*	1.64±0.08*
rWAT weight (g)	0.24±0.06	0.31±0.03	0.45±0.03*†	0.34±0.02*	0.40±0.02*
BAT weight (g)	0.15±0.01	0.21±0.03	0.24±0.02	0.19±0.01	0.24±0.05
Liver weight (g)	1.22±0.07	1.24±0.14	1.72±0.07	1.38±0.03	1.78±0.13*†
Serum level of					
AST (IU/L)	42.6±14.2	73.5±8.2	54.2±14.9	49.8±23.8	42.1±2.5
ALT (IU/L)	6.7±1.2	10.3±1.7	13.0±5.7	13.1±1.4	9.5±0.9
Glucose (mg/dL)	101.3±7.0	79.0±48.2	233.8±20.4†	112.7±14.9	264.1±57.8*†
TG (mg/dL)	126.0±19.2	111.0±6.6	96.1±10.1	89.6±6.5	75.8±9.4*
FFA (mEq/dL)	1.09±0.15	1.31±0.07	0.93±0.22	1.25±0.18	0.84±0.08

Data represent means ± SEM (3–5 mice). *$p < 0.05$ *vs.* baseline; †$p < 0.05$ *vs.* the LFD group at the same feeding time period. eWAT, epididymal WAT; rWAT, retroperitoneal WAT; AST, aspartate transaminase; ALT, alanine transaminase; FFA, free fatty acid.

Table S5
Body weight gains, tissue weights, and serum nutritional and biochemical parameters of HFD-fed C57BL/6J mice.

C57BL/6J	Baseline	20 weeks	
		LFD	HFD
Body weight gain (g)	–	11.1±0.8	22.3±4.1†
eWAT weight (g)	0.28±0.01	0.99±0.17*	1.53±0.17*†
rWAT weight (g)	0.06±0.00	0.29±0.05*	1.00±0.18*†
BAT weight (g)	0.07±0.01	0.12±0.01*	0.18±0.01*†
Liver weight (g)	0.90±0.01	1.10±0.04	1.56±0.2*†
Serum level of			
AST (IU/L)	36.2±3.9	38.8±2.6	56.3±8.7
ALT (IU/L)	12.7±3.6	3.8±0.6	28.1±8.9*†
Glucose (mg/dL)	113.5±5.0	88.0±11.6	179.4±26.4*†
TG (mg/dL)	118.9±16.7	84.1±7.7	87.8±21.2
FFA (mEq/dL)	1.40±0.08	1.06±0.10*	0.57±0.07*†

Data represent means ± SEM (8–9 mice). *$p < 0.05$ *vs.* baseline; †$p < 0.05$ *vs.* the LFD group at the same feeding time period. eWAT, epididymal WAT; rWAT, retroperitoneal WAT; AST, aspartate transaminase; ALT, alanine transaminase; FFA, free fatty acid.
